# Assessment of the ergogenic effect of caffeine supplementation on mood, anticipation timing, and muscular strength in older adults

**DOI:** 10.1002/phy2.72

**Published:** 2013-08-29

**Authors:** Jason Tallis, Michael J Duncan, Sheila Leddington Wright, Emma L J Eyre, Elizabeth Bryant, Dominic Langdon, Rob S James

**Affiliations:** Department of Biomolecular and Sport Sciences, Coventry UniversityJames Starley Building, Priory Street, Coventry, CV1 5FB, U.K

**Keywords:** Aging, anticipation, force, isokinetic, maximal voluntary contraction

## Abstract

The effect of caffeine to promote improvements in mood, cognition, and exercise performance has been well established in young and athletic adults. However, little is known about whether such nutritional ergogenic aids are effective in enhancing psychological well-being, physiological or cognitive performance in older adults. This study assesses the ergogenic effect of caffeine on mood, perceptual-motor coupling, and muscular strength in an older human population. Following a familiarization session, 12 apparently healthy volunteers (nine females and three males; 69 ± 6 years) completed two laboratory visits. “Pre ingestion” trials of mood state Brunel Mood State Inventory (BRUMS) and coincidence anticipation performance (Bassin anticipation timer) at slow (3 mph) and fast (8 mph) stimulus speeds were completed on both visits. Using a randomized, double-blind, cross-over design, participants consumed either caffeine (3 mg/kg body mass) or a placebo. Sixty minutes postingestion participants repeated the trials before completing a set of 10 consecutive repetitions of maximal knee extension using isokinetic dynamometry. Rating of perceived exertion (RPE) was assessed following the fifth and final repetition. Caffeine ingestion significantly improved mood state scores for vigor by 17% (*P* = 0.009) and reduced absolute error by 35% (*P* = 0.045) during coincidence anticipation assessment at 8 mph compared to placebo. There were no other significant effects. Caffeine ingestion failed to augment maximal voluntary contraction of the knee extensors and RPE did not prove to be significantly different to from placebo (*P* > 0.33 in each case). Acute caffeine ingestion may not be an effective ergogenic aid for improving muscular strength in older adults but could possibly be used as a nutrition supplement for enhancing mood and improving cognitive performance in daily living tasks where interceptive timing skills are required.

## Introduction

Caffeine is the most commonly consumed drug in the world and is widely used within sport and exercise settings for its performance enhancing effects, with evidence suggesting induced increases in endurance, strength, and power activities (Graham 2001; Davis and Green 2009). While the efficacy of acute caffeine ingestion for enhanced exercise performance is well established (Graham 2001), the majority of evidence demonstrating caffeine's ergogenic properties is derived from studies using participants that are well trained or within the range of peak physiological maturity (see reviews Graham 2001; Davis and Green 2009; Astorino and Roberson 2010). However, the ingestion of dietary caffeine is prevalent across the whole spectrum of society and with an increasingly aging population worldwide; authors have suggested that ergogenic aids such as caffeine may be useful in enhancing, performance during daily living tasks and psychological well-being in older adults (Cherniak 2012).

Exercise has often been used to increase mobility and enhance functional performance, leading to a decrease in falls and improved ability to undertake tasks of daily living (Goodpaster et al. 2008; Taguchi et al. 2010). One potential way to enhance exercise performance in older adults may be to provide adjunctive ergogenic aids (Cherniak 2012) with a range of substances including creatine, carnitine, and caffeine being reported in the literature. In younger adults, acute caffeine ingestion has been reported to dampen ratings of perceived exertion (Doherty and Smith 2005), enhance aerobic (Graham 2001), anaerobic, strength, and power (Astorino et al. 2010)-based exercise performance. Moreover, caffeine has been found to improve concentration (Nehlig 2010), attention, psychomotor performance (Nehlig 2010; Einother and Giesbrect 2013), with such changes being augmented as a result of exercise (Arciero and Ormsbee 2009; Duncan and Oxford 2011). However, data pertaining to the effect of caffeine on performance in older adults are scarce and merit further scrutiny (Cherniak 2012).

If such performance enhancing benefits can be elicited in an older adult population, acute caffeine ingestion might be one means by which to enhance any physiological or cognitive adaptations to exercise. Such adaptations may then lead to improved locomotor function and ability to undertake activities of daily living. Studies focusing on psychological and cognitive variables have documented improvements in simple and choice reaction times in 50- to 65-year olds (Swift and Tiplady 1988) following acute caffeine ingestion. One suggestion for these results is that caffeine ingestion may reverse the effects of cognitive aging by making greater energy reserves available in older adults (Lorist et al. 1995; Van Gelder et al. 2007) with some evidence demonstrating that such effects of caffeine ingestion may be more marked in older adults compared to younger adults (Swift and Tiplady 1988).

Compared with studies reporting on the effects of caffeine on cognitive and psychomotor performance in older adults, research examining acute caffeine ingestion on exercise performance in this population is sparse and equivocal. Norager et al. (2005) reported significant increases in cycling endurance (25%) and arm flexion endurance (54%), coupled with a reduction in rating of perceived exertion (RPE) in men and women aged over 70 years, following 6 mg/kg caffeine consumption, however, no significant differences in maximal isometric arm extension strength, walking speed, and reaction time were reported. Conversely, Momsen et al. (2010) demonstrated that 6 mg/kg of caffeine significantly increased maximal walking distance (20%) and maximal isometric knee extension strength (9.8%) in 68-year-old patients with moderate intermittent claudication. In agreement with the work of Norager et al. (2005), endurance was also significantly improved (21.4%), however, there was no effect of caffeine supplementation on reaction time and cognition. A later study by Jensen et al. (2011) adds further ambiguity after reporting that a 6 mg/kg caffeine supplement did not significantly improve maximal arm flexion strength and isometric submaximal endurance in subjects aged over 70 years.

The data in older adults have used caffeine doses ranging from absolute doses of 200 mg to relative doses ranging from 1.5 mg/kg to 9 mg/kg (Astorino et al. 2010; Einother and Giesbrect 2013). Caffeine doses in the range 3–6 mg/kg are considered to be optimum for ergogenic effects in both physical (Astorino et al. 2010) and psychological (Kenemans and Verbaten 1998; Brice and Smith 2001; Van Duinen et al. 2005) tasks while minimizing any adverse side effects of caffeine ingestion. With caffeine doses in past literature being varied, this study will examine the effect 3 mg/kg caffeine treatment, this dose is explicitly in the range of ergogenic doses identified in a review by Graham et al. (Graham 2001). The authors consider the motive for caffeine consumption in older adults to be markedly different to young athletic adults, whereby a performance enhancing benefit would have greatest value in aiding activities of daily life. 3 mg/kg caffeine is considered a closer representation of acute dietary caffeine, and an improvement seen at this level would have greater relevance in this population.

This study uses a sample of apparently healthy older adults to uniquely assess the effectiveness of a 3 mg/kg body mass dosage of caffeine on mood state and psychomotor performance at an increasing level of cognitive demand. Furthermore, the study aims to evaluate the effect of acute caffeine ingestion on maximal voluntary contraction of the knee extensor muscles in an older adult population. It is hypothesized that acute caffeine ingestion will elicit positive changes in mood state and coincidence anticipation time, which will be more greatly pronounced during instances of high cognitive demand. Furthermore, it is suggested that caffeine treatment will demonstrate small improvements in the ability to maintain short-term repeated maximal voluntary contraction of the knee extensor muscles.

## Method

### Participants

Following approval from the institutional ethics committee and informed consent, 12 volunteers (nine females and three males aged 61–79 years; 69 ± 2 years; height 166 ± 2 cm; body mass 73 ± 4 kg; body mass index (BMI) 26 ± 1 mean ± SE) agreed to participate. All participants were naive to resistance exercise training and all habitually ingested caffeine although none was a heavy caffeine user (all ingested less than 350 mg of caffeine per day; mean ± SE of caffeine consumption = 100.2 ± 9.3 mg/day). All participants completed a health history questionnaire to ensure that they met all inclusion criteria. Inclusion criteria were being “apparently healthy,” physically active, and accustomed to regular aerobic exercise. In this case, habitual physical activity was not directly assessed. However, participants were only eligible to participate if they reported engaging in 150 min of moderate physical activity or greater each week in line with recommendations for physical activity in older adults (BHF 2013). Participants were excluded if they had a musculoskeletal injury or cardiovascular condition which would restrict exercise performance, and who did not engage in 150 min of moderate physical activity per week or were a heavy habitual caffeine user. Participants were asked to abstain from caffeine intake and intense exercise for 48 h prior to each visit and undertook three visits to the human performance laboratory at Coventry University. In the first visit trials on the coincidence anticipation timer, isokinetic dynamometer and completion of the mood state questionnaire were conducted to familiarize participants with the equipment and procedures involved in the study. During this session, the anthropometric measurements were carried out in a private area in the laboratory prior to exercise. Prior to anthropometric measurements, participants were asked to remove shoes and any jackets worn. Height (cm) and mass (kg) were recorded to the nearest cm and 100 g, respectively, using a stadiometer (SECA Instruments, Ltd., Germany) and electronic weighing scales (SECA Instruments, Ltd.), respectively. BMI was calculated as kg/m^2^. In the following two experimental trials, participants completed measures of mood state and coincidence anticipation timing (CAT) (as a measure of cognitive performance) precaffeine and 60 min postcaffeine or placebo ingestion and also undertook assessment of isokinetic knee extension force. Each visit to the laboratory was separated by at least 72 h; subsequent trails were conducted at the same time of day as the initial visit in order to avoid any circadian rhythm effects on performance.

## Experimental Procedures

Following the familiarization session, participants completed “pre ingestion” trials on the coincidence anticipation timer and completed measures of mood state. Besides an enforced 48-h period of caffeine abstinence, preexperimental fed state was not directly controlled for; however, participants were given standardized instructions that they should consume their regular breakfast prior to testing on each day. Experimental trials were not conducted around typical meal times and subjects were in the laboratory approximately 1 h prior to ingestion of the treatment drink where no food was consumed. Participants were given either a caffeine treatment, where 3 mg/kg body mass of caffeine (Bayer, U.K.) diluted into 250 mL of artificially sweetened water was consumed, or a placebo where 250 mL of artificially sweetened water was consumed. Solutions were presented to participants in an opaque sports bottle to prevent the researchers administering the solutions or the participants from actually seeing the solutions themselves. Solutions were also matched for taste prior to the experimental protocols commencing. Solutions were administered double blind and consumed 60 min before each exercise trial as plasma caffeine concentration is maximal 1 h after ingestion of caffeine (Graham 2001). Participants undertook “post ingestion” trials on the coincidence anticipation timer, completed measures of mood state, and then completed measures of isokinetic knee extension.

### Mood state assessment

Mood state was assessed using the Brunel Mood State Inventory (BRUMS, Terry et al. 2003). This 24-item self-report measure is well established as a reliable and valid measure of mood state (Terry et al. 1999, 2003; Terry and Lane 2000, 2003) has been used in prior studies examining the effect of caffeine on mood (Duncan and Oxford 2011) and comprises subscales for anger, confusion, depression, fatigue, tension, and vigor. The coefficient of variation for BRUMS scores in older adults in our laboratory at rest is 0, 3.2, 3.5, 7.3, 5, and 9.1% for anger, confusion, depression, fatigue, tension, and vigor, respectively.

### CAT assessment

The Bassin anticipation timer (Model 35575, Lafayette, LA) was used to assess CAT performance in this study. The capacity to predict is an important underlying process in the performance of open skills (Rothstein and Wughalter 1987). CAT refers to the ability to predict the arrival of a moving object at a particular point in space and coordinate a movement response with that arrival (Payne 1986). As such it can be considered a test of perceptual-motor coupling requiring integration of sensory-cognitive procession and sensory-motor integration (Fleury and Bard 1985). Perceptual-motor coupling is fundamental to a multitude of actions within daily life including making judgments when crossing a busy street, walking through a crowd of shoppers, or catching a moving object (Sanders 2011).

During the familiarization session, participants were given 20 attempts at each of the stimulus speeds used in the study (3, 5, and 8 mph) to familiarize themselves with the test protocol. The Bassin Anticipation Timer was set up vertically in the front of the participant. Three sections of runway (2.24 m) were used, with the system's light-emitting diodes (LED) facing the participant. None of the lights on the runway was blanked and the target light was light #13. The sequentially lighted LED lamps illuminate in a linear pattern with movement occurring distally to proximally in front of the participant. For each trial, scores were recorded in milliseconds and whether the response was early or late. The start and end speeds remained constant at 3, 5, and 8 mph for all trials to represent slow and fast stimulus speeds, congruent with prior research (Duncan et al. 2013). Presentation of stimulus speeds was randomized and counterbalanced. To reduce the likelihood that the participant could internally time the trial, cue delay (visual warning system) was set as random on the timer with a minimum delay of 1 sec and a maximum delay of 2 sec. For each trial, the signal was initiated by the experimenter, with the participant being asked to press a trigger button, with their dominant hand, as close to the arrival time of the stimulus at the target location as possible. Participants completed 10 trials at each stimulus speed per trial. Raw scores across each of the stimulus speeds were summarized into three error scores, constant, variable, and absolute, as a means of generating the dependant variables. This approach is consistent with recognized protocols using CAT scores (Isaacs and Pohlman 1991; Lyons et al. 2008; Sanders 2011). The coefficient of variation for CAT at 3 and 8 mph in older adults in our laboratory at rest is 7.1 and 7.9%, respectively.

#### Constant error

The temporal interval (in milliseconds) between the arrival of the visual stimulus and the end of the participant's motor response; It represents the mean response of an individual and the direction of error: early or late (Schmidt 1982).

#### Variable error

The participant's standard deviation from their mean response; this represents the variability/inconsistency of responses.

#### Absolute error

The value of participant's raw scores disregarding whether the response was early or late.

### Isokinetic knee extension strength assessment

Postingestion of the given treatment, and directly following CAT assessment, measurement of maximal voluntary strength of the knee extensors of the dominant leg was evaluated using isokinetic dynamometry (KinCom 125AP; Chattanooga Group, Chattanooga, TN). A systematic review by Warren et al. (2010) concluded that caffeine-induced strength improvements appear exclusively in the knee extensors. Astorino et al. (2011) recently reported caffeine-mediated improvements in intense resistance training of the legs that were not paralleled in upper body exercise in a young adult population; hence, assessment of knee extensor strength was used in this study.

Prior to this assessment, the inbuilt dynamometer warm up feature was used in order to minimize injury risk and to ensure that participants were primed for the exercise protocol. The participants were then asked to complete a set of 10 consecutive repetitions of maximal knee extension through 70°, at a contraction velocity of 30°/sec. Participants were given strong verbal encouragement throughout both trials, but were not given any feedback about their performance during the protocol. Peak and average force produced were recorded for each repetition. Cumulative peak force, the sum of the peak force achieved over the 10 repetitions, and cumulative average force, the sum of the average force achieved over the 10 repetitions, were then calculated as a measure of fatigue. RPE (Borg 20-point scale) (Borg 1970) was also recorded following fifth and 10th repetition.

### Statistical analysis

A series of 2 (Pre- to Postingestion) × 2 (Caffeine vs. Placebo) ways repeated measures analysis of variance were conducted to examine any differences in constant error, variable error, and absolute error on the CAT task, and to test for any differences in each of the subscales on the BRUMS. Where significant differences were found, Bonferroni post hoc pairwise comparisons were used to determine where the differences lay. Partial eta squared (*η*^2^) was used as a measure of effect size. Paired *t*-tests were used to detect differences in maximal voluntary knee extensor strength between caffeine and placebo trials. A further two-factor analyses of variance (ANOVA) was used to examine any differences in RPE over time (Repetition 5 vs. 10) and between conditions (Caffeine vs. Placebo). The Statistical Package for Social Sciences (SPSS, Version 20, Chicago, IL) was used for all analyses and statistical significance was set, a priori, at *P* = 0.05. Data are reported as mean ± SE.

## Results

### Coincidence anticipation timing

There were no significant main effects (either prepost or due to the substance ingested) or interactions (between substance and main effect) for CAT responses at slow (3 and 5 mph) stimulus speeds (all *P* > 0.05) for absolute, constant, or variable error.

**Figure 1:**
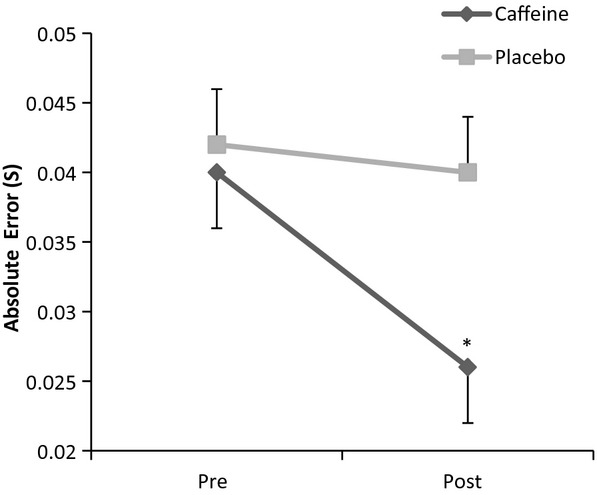
Caffeine ingestion reduced absolute error in coincidence anticipation timing in older adults (data represented as mean ± SE; *significant statistical differences; *n* = 12).

For coincidence anticipation responses at fast stimulus speeds (8 mph), there was a significant substance X prepost interaction for absolute error at 8 mph (*F*_1,12_ = 5.013, *P* = 0.045, partial η^2^ = 0.295; Fig. 1). There were no significant differences in constant or variable error (*P* > 0.05). Bonferroni post hoc pairwise comparisons indicated that there was a significant (*P* = 0.045) 35% reduction in absolute error (i.e., an improvement) following ingestion of caffeine. No improvement in CAT was evident in the case of the placebo condition.

### Mood state

**Figure 2:**
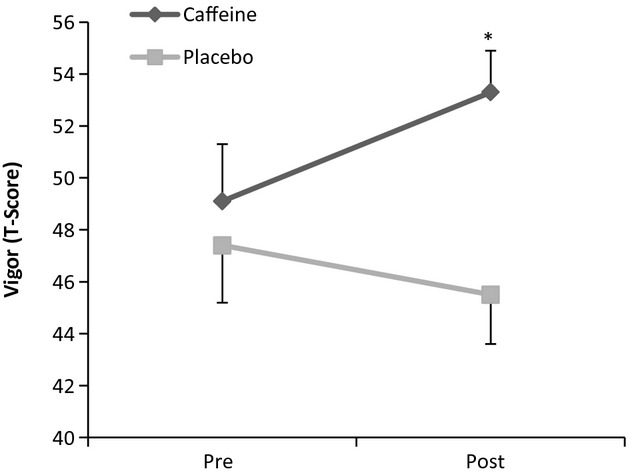
Caffeine enhance mood state scores for vigor (T-Score) in older adults (data represented as mean ± SE; *significant statistical differences; *n* = 12).

There were no significant main effects or interactions (all *P* > 0.05) for responses on the anger, confusion, depression, tension, and fatigue subscales of the BRUMS. However, for the vigor subscale, results indicated a significant substance X prepost interaction (F_1,12_ = 9.651, *P* = 0.009, partial η^2^ = 0.446) with standardized scores for vigor significantly increasing by 17% postcaffeine ingestion compared to postplacebo ingestion (Fig. 2.)

### Muscle strength and perception of effort

**Figure 3:**
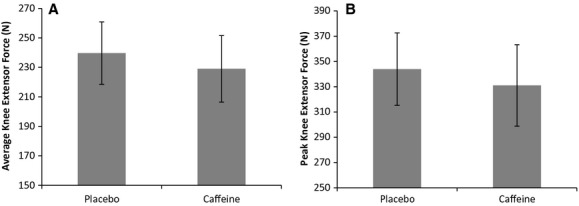
Acute caffeine ingestion did not affect average (A) or peak (B) isokinetic knee extension force in older adults (data represented as mean ± SE; *n* = 12).

**Figure 4:**
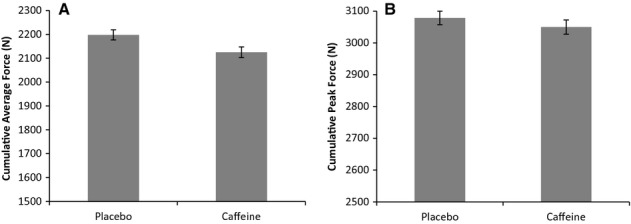
The effect of an acute caffeine ingestion on cumulative average (A) and peak (B) isokinetic knee extension force after 10 repetitions (data represented as mean ± SE; *n* = 12).

There was no effect of acute caffeine intake on maximal average isokinetic knee extension force when compared to placebo (Fig. 3A, *P* = 0.733). Similarly, there was no effect on maximal peak isokinetic knee extension force in the caffeine trial (Fig. 3B, *P* = 0.767). Furthermore, caffeine supplementation failed to augment cumulative average force and cumulative peak force when compared to placebo (Fig. 4A, *P* = 0.816; Fig. 4B. *P* = 0.946, respectively). RPE after five repetitions was 14.92 ± 0.71 and 15.08 ± 0.71, and after 10 repetitions 15.42.92 ± 0.71 and 15.92 ± 0.71 for the caffeine and placebo trial, respectively. RPE did not prove to be significantly different between the caffeine and placebo trial (*P* = 0.638), nor was the effect significant over time (*P* = 0.631).

## Discussion

This study demonstrates that acute caffeine ingestion (3 mg/kg) failed to elicit a significant change in maximal voluntary peak and average muscle force in this sample of older adults. However, we did find that caffeine ingestion enhanced CAT performance at fast stimulus speeds and favorably changed mood state to exercise when compared to placebo.

### Mood and timing performance

The results reported here in relation to mood state changes add support to prior studies in young adults which have also reported similar enhanced mood state following ingestion of caffeine (Arciero and Ormsbee 2009; Duncan and Oxford 2011). However, although Nehlig (2010) asserted that caffeine ingestion positively influences mood state, this effect on mood was thought to be amplified in older compared to younger adults. In our present findings, the only subscale of the BRUMS which evidenced any significant difference was on scores for vigor. This is not surprising as it is one of the main facets of mood which is consistently shown to be influenced by caffeine ingestion (Smith 2002). However, in other work, different subscales have changed as a result of caffeine ingestion, particularly scores for tension and vigor (Arciero and Ormsbee 2009; Duncan and Oxford 2011). The effects of caffeine on mood are, however, postulated to be more apparent in conditions of low arousal (Smith 1994; Smith and Tola 1998) particularly in the workplace or during repetitive tasks. In this study, it is likely that the participants' unfamiliarity with laboratory-based experiments may have contributed to increased levels of physiological arousal, even at rest and as such we speculate that any large changes in mood state as a result of caffeine ingestion may have been masked.

Coincidence anticipation timing contributes to success in numerous tasks of daily living, but particularly where predicting the arrival of a moving object is important, for example, crossing a busy street (Sanders 2011). The results of this study are novel as they evidence that caffeine ingestion may enhance CAT performance compared to placebo ingestion, but only at fast stimulus speeds. These results add support to prior claims that caffeine ingestion may enhance performance particularly where cognitive and/or perceptual-motor skills are important (Kenemans and Verbaten 1998; Brice and Smith 2001; Van Duinen et al. 2005). To some extent the positive impact of caffeine ingestion on such performance is not unexpected. Caffeine ingestion has measureable performance-enhancing abilities (Jones 2008). This is, in part, due to its impact on the central nervous system (CNS; Kalmar and Cafarelli 2004) through adenosine inhibition (Keisler and Armsey 2006). However, no previous studies appear to have examined whether caffeine ingestion influences CAT performance in older adults. In this respect, our data are significant as older adults have been reported to be comparatively more affected by increased stimulus velocity in perceptuo-motor tasks than younger adults (Haywood 1980; Meeuwsen et al. 1997). Moreover, improving perceptual-motor coupling performance has been identified as key in reducing accidents in older adults (Stelmach and Nahom 1992), a population at greater risk of accidents during tasks of daily living (Lobjos et al. 2006). As there are no studies on older adults reporting the impact of caffeine ingestion on coincidence timing performance specifically, it is difficult to compare the results presented here with prior research. However, one study by Lobjos et al. (2006) reported the effect of tennis playing experience on CAT performance. They reported that older, nontennis playing adults had significantly poorer CAT performance at fast stimulus speed (similar to that used in this study) represented by scores for variable and constant error. Surprisingly, Lobjos et al. (2006) did not report scores for absolute error although this is the more commonly accepted measure of timing error in the literature (Lyons et al. 2008; Sanders 2011). In this study, an ergogenic effect of caffeine on timing performance was only seen at fast stimulus speed. There are some explanations for this. As the timing tasks in this study were performed at rest, the participants may have been less physiologically aroused during the slow stimulus speed trials compared to the fast stimulus speed trials. The effect of caffeine on the CNS may therefore have allowed greater attention capacity on the fast stimulus speed trials augmenting the effect of a higher physiological arousal level (compared to the slow stimulus speed trials) and producing enhanced timing performance. Such a suggestion is speculative although congruent with Kahneman's 1973 multidimensional allocation of resources theory. Within this theory, performance at higher task difficulty will deteriorate, as cognitive effort cannot focus attention solely on task-relevant information (Kahneman 1973). High and low levels of physiological arousal can cause the individual to direct attention to many different sources, some of which provide irrelevant information and cause relevant signals to be missed. An example of this, when exercise is the stressor would be the inability to ignore perceptions of pain, distress or fatigue (McMorris and Keen 1994).

Caffeine has been established to effectively increase CNS activation (Kalmar and Cafarelli 2004), the potential of this coupled with positive changes in mood state in the caffeine trails may have resulted in enhanced performance at higher levels of task difficulty compared to the placebo condition.

### Muscle strength and perception of effort

The present findings indicate that caffeine does not increase maximal knee extensor strength in older adults. This is concurrent with previous findings in similar populations where a higher dose of caffeine (6 mg/kg) had no effect on maximal isometric arm extension (Norager et al. 2005; Jensen et al. 2011). Previous work examining the ergogenic properties of caffeine in the elderly have demonstrated equivocal results when measuring submaximal isometric muscle endurance (50–60% of maximal) (Norager et al. 2005; Momsen et al. 2010; Jensen et al. 2011). This study is the first to assess whether caffeine treatment will effectively enhance maximal voluntary isokinetic muscle contraction, and ability to maintain maximal effort, in an older adult population. The present findings further indicate that caffeine treatment failed to augment cumulative force production over 10 repetitions. This evidence therefore suggests that caffeine is not an effective ergogenic aid for improving muscle strength in older adults.

In contrast to the present results, caffeine has usually been found to elicit an increase in muscular strength in a younger population (Astorino and Roberson 2010; Astorino et al. 2010). Astorino et al. (2010) demonstrated that 5 mg/kg acute caffeine supplementation significantly increased isokinetic knee extensor strength in 26-year-old participants. Furthermore, a recent study by Pallarés et al. (2013) examining the effect of an incremental caffeine treatment on muscle force and power in young trained men, concluded that the caffeine dose required to be ergogenic is dependent on the magnitude of load used. Caffeine-induced improvements in performance at lighter loads were maximized with a lower dose of caffeine (3 mg/kg) and in contrast, higher doses were needed to evoke performance enhancements at higher loads (9 mg/kg for 90% one repetition maximum).

Therefore, it is considered that 3 mg/kg caffeine may also be insufficient in improving high-intensity strength performance in the present population. Further inconsistencies with previous research demonstrating positive effects of caffeine on muscular strength may also relate to an age-related decline in the performance-enhancing effect. Consideration of the mechanism for this reduction in ergogenic benefit should be explored. Initially, it is considered that caffeine is a habitual drug and regular consumption will reduce the magnitude of its effects. Bell and Mclellan (2002) demonstrated that an acute supplementation of caffeine significantly improved endurance capacity to a higher degree in participants who were noncaffeine users. Although not deemed to be heavy caffeine users (<350 mg/day), participants habitually ingested caffeine (100.2 ± 25.2 mg/day), which may rationalize the absence of an ergogenic effect in the tested population.

Previous animal work has highlighted that caffeine may increase power by acting directly at the skeletal muscle (Tallis et al. 2012; Tallis 2013). Later evidence by the authors using an animal model has suggested that caffeine may still provide positive ergogenic properties in extensor digitorum longus and diaphragm muscle from older mice (50 weeks), but that this effect is significantly reduced compared to a younger age group (10 weeks; Tallis 2013). This is likely to be related to sarcopenia and more specifically an age-related reduction in the number of physiologically active Ryanodine receptors (Delbono et al. 1995; Renganathan et al. 1997). Consequently, the action of caffeine to modulate intramuscular calcium concentration would be significantly reduced and subsequently the effectiveness of caffeine to work directly at the skeletal muscle is impaired. A combination of habituation and the age-related deterioration of skeletal muscle are likely to contribute to the reduced effectiveness of caffeine as an ergogenic aid in a more senior population.

Although caffeine has been reported to effectively modulate perceptual responses in younger adults during prolonged exercise (Doherty and Smith 2005), the effect of caffeine to reduce the perception of effort was not demonstrated in this study and is continuous with the findings of Jensen et al. (2011), who also reported no difference in RPE in a similar aged population undertaking moderate intensity cycling. Our findings further suggest that caffeine is not effective at reducing the perception of effort during short duration, high-intensity activity in an elderly population. In support of previous literature on younger participants, it appears that caffeine does not alter RPE during or at completion of short-term, high-intensity exercise (Jacobs et al. 2003; Astorino et al. 2008, 2010; Astorino and Roberson 2010; Duncan and Oxford 2011).

### Limitations and future work

In order to contextualize the broader application of these findings the limitations to this study should be considered. Initially, the 3 mg/kg concentration of caffeine administered to the participants is a relatively mild dose and research in younger adults indicates that higher doses of caffeine may be needed to elicit positive changes in muscle strength (usually around 5–6 mg/kg; Norager et al. 2005; Astorino et al. 2008; Astorino and Roberson 2010; Momsen et al. 2010; Jensen et al. 2011). With this in mind, a study comparing the ergogenic effects of caffeine in older adults compared to younger adults would be an interesting area of future research. Furthermore, the method of exercise to examine muscular strength is not representative of dynamic muscular contraction in daily activities and was only measured at a single contraction speed. As with this study, previous research examining the ergogenic properties of caffeine in an older population (Norager et al. 2005; Momsen et al. 2010; Jensen et al. 2011) has used assessments of maximal voluntary contraction to evaluate muscular strength. The authors recognize that the assessment of peak force may not accurately reflect the strength requirements of tasks of daily living in older adults. Future research may consider using an adapted protocol that closely represents the strength requirements of this population. Furthermore, due to the relatively strict inclusion criterion for this study, we were unable to control for training status and caffeine habituation. In consideration of this, examining the effects of caffeine withdrawal in the present work would have been an interesting addition. Future research may consider evaluating the effect of caffeine in nonhabitual older adults, although this may be difficult in a western population.

The methods employed in this study are a useful first step for assessing perceptual-motor coupling and the Bassin anticipation timer was employed as this is the most widely validated measure of coincidence anticipation currently available (Sanders 2011). However, the authors consider employing more specific coincident timing protocols (e.g., simulating crossing a road) and examining coincidence anticipation responses to exercise using nonuniform and nonlinear motion would be a useful area of future research.

## Conclusion

There is no ergogenic effect of a relatively low dose of caffeine to promote enhancements in muscular strength and modification in effort perception during efforts requiring maximal voluntary contraction in a more senior population. Caffeine, however, may still have positive central effects in older adults inducing enhancements in mood and CAT performance where a fast stimulus speed is involved. Thus, caffeine may prove beneficial to task of daily living where interceptive timing skills are required.
